# Corticosteroid Use and Long-Term Changes in Weight and Waist Circumference: The Lifelines Cohort Study

**DOI:** 10.1210/clinem/dgaf166

**Published:** 2025-05-01

**Authors:** Mostafa Mohseni, Eline S van der Valk, Maartje J B Van der Hurk, Mesut Savas, Mariëtte R Boon, Elisabeth F C van Rossum

**Affiliations:** Obesity Center CGG, Erasmus MC, University Medical Center Rotterdam, P.O. Box 2400, 3000 CA Rotterdam, the Netherlands; Division of Endocrinology, Department of Internal Medicine, Erasmus MC, University Medical Center Rotterdam, P.O. Box 2400, 3000 CA Rotterdam, the Netherlands; Obesity Center CGG, Erasmus MC, University Medical Center Rotterdam, P.O. Box 2400, 3000 CA Rotterdam, the Netherlands; Division of Endocrinology, Department of Internal Medicine, Erasmus MC, University Medical Center Rotterdam, P.O. Box 2400, 3000 CA Rotterdam, the Netherlands; Obesity Center CGG, Erasmus MC, University Medical Center Rotterdam, P.O. Box 2400, 3000 CA Rotterdam, the Netherlands; Division of Endocrinology, Department of Internal Medicine, Erasmus MC, University Medical Center Rotterdam, P.O. Box 2400, 3000 CA Rotterdam, the Netherlands; Obesity Center CGG, Erasmus MC, University Medical Center Rotterdam, P.O. Box 2400, 3000 CA Rotterdam, the Netherlands; Division of Endocrinology, Department of Internal Medicine, Erasmus MC, University Medical Center Rotterdam, P.O. Box 2400, 3000 CA Rotterdam, the Netherlands; Obesity Center CGG, Erasmus MC, University Medical Center Rotterdam, P.O. Box 2400, 3000 CA Rotterdam, the Netherlands; Division of Endocrinology, Department of Internal Medicine, Erasmus MC, University Medical Center Rotterdam, P.O. Box 2400, 3000 CA Rotterdam, the Netherlands

**Keywords:** glucocorticoids, body weight, waist circumference, corticosteroids

## Abstract

**Context:**

The use of corticosteroids (CS) has been associated with higher body mass index (BMI) and waist circumference (WC) in cross-sectional studies. However, longitudinal data are scarce, particularly for locally administered forms.

**Design:**

We analyzed weight and waist circumference changes in 81 361 Lifelines Cohort Study participants (mean age 46.3 years, mean BMI 26.0 kg/m^2^, 41% male, mean follow-up 3.9 years) via linear regression. Sensitivity analyses included stratification by sex and BMI. Short-term weight changes post-start were assessed in a subset using linear mixed-effect models.

**Results:**

We found 23.8% CS users during the study period. Individuals reporting any new use of CS gained significantly more weight compared to nonusers at follow-up (β .034 kg/year, *P* = .021), particularly among those initiating local CS use (β .037 kg/year, *P* = .017). Use of new systemic CS was associated with increased WC (β .200 cm/year, *P* < .001). Discontinuation of CS led to decreased WC (β −.078 cm/year, *P* = .028). These effects were particularly observed in female participants and individuals with BMI ≥25 kg/m^2^, but not in male participants and those with BMI < 25 kg/m^2^. Short-term weight-inducing effects of CS were not observed in the weeks after initiation of CS use.

**Conclusion:**

This study demonstrates that CS use, including locally administered forms, is associated with long-term increases in weight and WC, notably in female individuals and those with overweight or obesity. Discontinuing CS was linked to reductions in WC. These findings underscore the need to carefully assess chronic systemic and local CS use, as discontinuation could benefit obesity-related outcomes in certain patients.

Over the last decades, the prevalence of overweight and obesity has dramatically increased, resulting in significant obesity-related morbidity and mortality (1). Obesity is a chronic and progressive disease (2), usually with a multifactorial etiology where lifestyle, medication use, endocrine, psychosocial, and genetic factors can play a role in the development and maintenance of this condition (3, 4).

Corticosteroid-containing drugs, in all possible administration forms, are frequently mentioned as possible contributing factors to development and maintenance of obesity (5).

Corticosteroids (CS) are anti-inflammatory drugs used for the treatment of a myriad of diseases. The number of conditions for which CS are indicated has been increasing, and consequently, the prescription rates of these drugs have risen as well (6, 7). Several studies have shown that the use of local CS is also associated with obesity and metabolic syndrome in adults (8, 9). Recent studies indicate that inhaled CS induces adrenal suppression of glucocorticoid hormone production in a dose-dependent manner, suggesting systemic effects of locally administered CS (10, 11). Although in pediatric cohorts it has been shown that the use of inhaled CS is related to greater body mass index (BMI) increase over time (12), long-term data on the association of (local) CS use and weight changes in the general adult population is scarce.

In this study we aimed to investigate the longitudinal association of chronic systemic and local CS use on changes in both weight and waist circumference with a follow-up period of 5 years. In addition, we evaluated whether also any short-term (12 weeks after initiation) associations could be found.

## Methods

### Study Design and Population

Individuals participating in the Lifelines cohort study were included in this study. The overall design and rationale of Lifelines has been extensively described elsewhere (13, 14). In brief, Lifelines is a 3-generation, prospective, population-based cohort study. Lifelines aims to investigate risk factors for diseases in multiple generations with a long-term follow-up. Lifelines assesses a range of biomedical, socio-demographic, behavioral, physical, and psychological factors which contribute to health and disease. Individuals living in the northern provinces of the Netherlands and who were registered with a general practitioner (GP) were eligible to participate. Exclusion criteria for participation in Lifelines were: severe psychiatric or physical illness, limited life expectancy (<5 years), and insufficient knowledge of the Dutch language to complete a Dutch questionnaire.

Baseline data (T1) of these 167 729 participants were collected between 2007 and 2013. Participants are followed up every 5 years, and the second assessment (T2) of this cohort took place between 2014 and 2017. A total of 81 361 participants who participated in both T1 as well as T2 assessment, and therefore had weight reported at both time points, were included for analysis.

For the Lifelines cohort study, written informed consent was provided by participants. The Lifelines protocol was approved by the Medical ethical committee of the University Medical Center Groningen under number 2007/152. The Lifelines database was created using the open source MOLGENIS software 9.2.3, built on 2022-04-01 (15).

### Assessment of Corticosteroid Drug Use and Anthropometric Measurements

At T1, drug use was evaluated using a self-reported questionnaire and by asking participants to bring their used medications at the time of baseline assessment for inspection by the research nurse. All prescribed medications were classified according to the World Health Organization Anatomical Therapeutic Chemical (ATC) coding scheme. We classified CS use into systemic CS (ie, oral or parenteral CS) or local CS (ie, dermal, intranasal, inhaled, ontological, ocular, local oral, and gynecological forms).

At T2, use of CS was evaluated using questionnaires, which were filled out in the context of hair glucocorticoid analysis applying to the 3 months prior to the study visit. Similar to T1, CS use was classified into systemic and local CS use. Multiple questions assessed different aspects of medication use; therefore CS use and subsequently systemic and local CS were assessed by making dummy variables for systemic CS use or local CS use on either T1 or T2 by compounding the answers to the questions in Supplemental data 1 (16).

At T1 and T2, measurement of weight and waist circumference (WC) were performed consistently following standardized operating protocols by trained research nurses. Body weight (in kg) and height (in cm) were measured without shoes and accurately to the nearest half unit. BMI was calculated by dividing body weight by height in meters squared. Waist circumference was measured in an upright position and in the middle between the front edge of the lower ribs and the iliac crest.

### Assessment of Covariates

By using questionnaires, we obtained self-reported data concerning educational attainment (classified as low/medium/high according to the standardized cutoffs applicable to the Dutch education system; in Dutch: “Standaard onderwijsindeling”), alcohol use was classified into “non-drinkers,” “0-1 drinks per day,” “1-2 drinks per day,” or “more than 2 drinks per day.” Smoking was categorized into “current smoking,” “never smokers,” and “quit smoking.”

Comorbidities were assessed at T1 and T2, but also using interim questionnaires which were sent out in between T1 and T2. A compound variable was created to assess whether participants reported any prevalent cardiovascular diseases (CVD), which included a medical history of the following conditions: heart failure, myocardial infarction, coronary artery bypass graft (CABG) or percutaneous coronary intervention (PCI), peripheral arterial vascular disease, cerebrovascular disease, cardiac arrhythmia (diagnosed by a physician), and aortic aneurysm.

Furthermore, compound variables were created whether participants report the presence of either type 1 or type 2 diabetes mellitus, cancer, and asthma or chronic obstructive pulmonary disease (COPD) at either T1, T2, or interim questionnaires.

### Substudy Short-term Follow-Up

Short-term effects of CS use on weight were assessed in the Lifelines COVID-19 cohort, which is a subcohort of participants of Lifelines who responded to the invitation to fill out questionnaires during the first wave of the COVID-19 pandemic from April 2020 to June 2020 (13). Questionnaires were sent out weekly during the first 9 weeks, and biweekly thereafter. A total of 60 956 participants who had reported weight at least 2 time points (mean 7.3 responses; range 2-11) were included in our analyses. Weight and corticosteroid use, alcohol use, smoking, physical activity, and meal frequency was self-reported using a questionnaire. Similar to the T1-T2 analyses, comorbidities were assessed using the same compound variables for CVD, cancer, diabetes mellitus, and asthma/COPD, but with addition of the questionnaires sent out during the COVID-19 pandemic.

### Statistical Analysis

Data was checked for normality. Descriptive statistics of the baseline measurements are reported as median and interquartile range (IQR) for non-parametric data and mean ± SD for parametric data. Differences in baseline characteristics between CS users and non-CS users were analyzed using Student *t* test for continuous variables, and chi-square test for categorical data.

Weight change (kg/year) and change in WC (cm/year) were calculated using the absolute difference in weight and WC between T1 and T2, and outliers were excluded from analyses (defined as > ±3 SD from the mean difference in weight or WC, as it is unlikely that these cases represent the normal course of weight, but are likely influenced by intercurrent conditions). Multivariable linear regression models were used to assess the effect of CS use on weight and WC change between T1 and T2. Model A shows a crude analysis for the association between change in weight or WC and the use of CS. Model B, the association between change in weight or WC and the use of CS was adjusted for age, sex, baseline BMI, native Dutch ethnicity, educational level, smoking status, and alcohol use. Model C included the covariates used in model B, in addition to comorbidities (ie, CVD, cancer, diabetes mellitus, and asthma/COPD).

For the analyses assessing short-term changes in weight, based on visual inspection of the data, discrepant self-reported weights (ie, <38 kg and >230 kg) were excluded from analyses. Participants who reported (extreme) weight changes of >10 kg within 1 week were also excluded from analyses. A linear mixed-effects model was used to model individual weight trajectories and assess whether corticosteroid use had an effect on weight change during the follow-up period of 12 weeks. Fixed effects included corticosteroid use, age, sex, ethnicity, comorbidities, alcohol use, smoking, meal frequency, physical activity. Estimates for the interaction between time (in weeks) × CS use are reported, resulting in an estimation of weight change per week determined by CS use.

A significance level of *P* < .05 was used. The statistical analyses were performed using IBM SPSS Statistics version 28.0.1.1 (SPSS Inc.) and R Studio version 2022.02.0 (build 433).

### Sensitivity Analyses

To explore the effects of CS use on sex and different BMI classes, we repeated the analyses in males and females separately and also repeated the analyses after stratifying cases into normal weight and overweight, defined as respectively BMI <25 kg/m^2^ and BMI ≥25 kg/m^2^.

## Results

### Baseline Characteristics

Of the 81 361 individuals who were included in the analyses (Table 1), mean time to follow-up was 3.9 years (SD 1.2 years), and 19 424 participants (24%) reported any use of CS at either baseline (T1) or at follow-up (T2). Table 2 shows the use of CS in our cohort. Local CS use was reported by 18 292 participants. This included 7183 participants using inhalation corticosteroids, 7933 using intranasal corticosteroid sprays, and 6362 using topical corticosteroids (ie, dermal, hemorrhoidal, or oral creams and ointments, eye- and ear-drops). The majority of participants (59.2%) were female and native Dutch. Mean age of the entire cohort was 46.3 years old, with a mean weight of 79.3 kg (BMI 26.0 kg/m^2^) and mean WC of 90.2 cm (95.4 cm for males and 86.8 cm for females). At T1, individuals who reported any CS use had significantly higher weight, BMI, and waist circumference (mean difference 0.83 kg, 0.62 kg/m^2^, and 1.6 cm, respectively; all *P* < .001) compared to individuals who never reported any use of CS.

**Table 1.** Baseline characteristics

	N	No CS use (n = 61 937)	Any CS use (n = 19 424)
Age (years)	81 361	46.3 (±12.6)	46.6 (±13.0)**
Sex, female	81 361	36 671 (59.2%)	12 321 (63.4%)***
Ethnicity, native Dutch	81 098	58 984 (95.5%)	18 363 (94.9%)***
Education	79 536		
Low		18 231 (30.1%)	6257 (33.0%)***
Medium		23 763 (39.2%)	7296 (38.5%)***
High		18 581 (30.7%)	5408 (28.5%)***
Comorbidities			
Asthma	81 175	2509 (4.1%)	4638 (24.0%)***
COPD	80 958	1399 (2.3%)	3074 (16.0%)***
Myocardial infarction	81 072	610 (1.0%)	232 (1.2%)*
Aortic aneurysm	81 095	151 (0.2%)	69 (0.3%)**
PCI or CABG	80 952	833 (1.4%)	336 (1.7%)***
Heart failure	80 378	400 (0.7%)	174 (0.9%)***
Stroke	80 920	433 (0.7%)	169 (0.9%)*
Hypertension	79 902	13 141 (21.6%)	4763 (25.1%)***
Cancer	81 237	2892 (4.7%)	1047 (5.4%)***
Diabetes mellitus	81 193	1359 (2.2%)	615 (3.2%)***
Physical activity	76 660		
0 days/week		2664 (4.6%)	875 (4.8%)
1-4 days/week		26 618 (45.4%)	8297 (45.7%)
5-7 days/week		29 220 (50.0%)	8986 (49.5%)
Smoking status	77 464		
Current smoker		11 336 (19.2%)	3554 (19.2%)***
Former smoker		19 922 (33.8%)	6608 (35.6%)***
Nonsmoker		27 665 (47.0%)	8379 (45.2%)***
Alcohol use	81 361		
No		10 258 (16.6%)	3387 (17.4%)***
<1 day/week		13 314 (21.5%)	3907 (20.1%)***
1-3 days/week		24 420 (39.4%)	6519 (33.5%)***
4-7 days/week		13 870 (24.4%)	3776 (19.4%) ***
Weight (kg)	81 361	79.15 (±14.71)	79.98 (±15.54)***
BMI (kg/m^2^)	81 361	25.86 (±4.07)	26.48 (±4.60)***
Waist circumference (cm)	81 361	89.8 (±12.0)	91.4 (±12.8)***
Systolic blood pressure (mmHg)	81 321	126 (±15)	126 (±15)
Diastolic blood pressure (mmHg)	81 321	74 (±9)	74 (±9)
Weight change T2-T1 (kg/year)	78 126	0.056 (1.062)	0.078 (1.096)*
Waist circumference change T2-T1 (cm/year)	80 373	0.023 (1.727)	0.048 (1.767)

Baseline characteristics of the study sample, stratified by corticosteroid use at either baseline (T1) or second assessment (T2; mean follow-up time 3.9 years), and no CS use at T1 or T2. Data presented as mean ± SD, or n (%). *P < .05, **P < .01, ***P < .001.

Abbreviations: BMI, body mass index; CABG, coronary artery bypass graft; COPD, chronic obstructive pulmonary disease; PCI, percutaneous coronary intervention.

**Table 2.** Corticosteroid drug use at baseline and follow-up

	Any CS use (n)	Systemic CS use (n)	Local CS use (n)
No CS use at T1 or T2	61 937 (76%)		
CS use at either T1 or T2	19 424	2351	18 292
Discontinuation at follow-up	5540 (7%)	372	5448
New CS use	8867 (11%)	1812	8130
CS use at both T1 and T2	5017 (6%)	167	4714

“Any CS use”: either systemic CS use or local CS use.

“Discontinuation at follow-up”: CS use reported at T1, but not at T2.

“New CS use”: CS use only reported at T2, not at T1.

Abbreviations: CS, corticosteroid; T1, baseline assessment; T2, second assessment.

### Long-Term Changes in Weight and Waist Circumference, and Reported CS Use

Long-term associations of CS use and changes in weight and WC were assessed in the full cohort. At T2, individuals reporting CS use at either baseline or follow-up had a higher rate of weight gain (mean difference 0.022 kg/year; *P* = .014) and a tendency toward an increase in WC (mean difference 0.025 cm/year, *P* = .076) compared to individuals who had never reported any CS use (Table 1).

### Long-Term Changes in Weight and Waist Circumference in New CS Users

Table 2 shows the number of participants who were new users, discontinuers, or continued users, within the groups of any CS use, systemic CS use, and local CS use. Table 3 reports the regression estimates for weight change between T1 and T2. New use of any CS (ie, no CS use at T1, but only CS use at T2) was associated with higher weight gain compared to never users of CS (β .034 kg/year, *P* = .021, Table 2). When analyzing systemic CS users and local CS users separately, this association was only seen in new local CS users (β .037 kg/year, *P* = .017), although this trend was also observed in new use of systemic CS (β .055 kg/year, *P* = .084). In participants with continued systemic CS use (ie, CS use at both T1 and T2) we saw a trend toward weight loss compared to never users (β −.169 kg/year, *P* = .082).

**Table 3.** Linear regression estimates for weight change between T1 and T2

	Model A—crude analyses			Model B—baseline characteristics			Model C—comorbidities		
	N	beta (95% CI)	*P* value	N	beta (95% CI)	*P* value	N	beta (95% CI)	*P* value
Any CS use	76 285			66 012			56 656		
Discontinuation at follow-up		−0.030 (−0.067 to 0.007)	.110		−0.014 (−0.053 to 0.026)	.491		−0.026 (−0.069 to 0.017)	.229
New CS use		**0.049 (0.024 to 0.073)**	**<**.**001**		**0.045** (**0.019 to 0.072)**	.**001**		**0.034** (**0.005 to 0.063)**	.**021**
CS use at T1 and T2		0.005 (−0.026 to 0.037)	.752		0.034 (−0.000 to 0.068)	.050		0.019 (−0.022 to 0.059)	.364
Systemic CS use	61 813			53 436			46 097		
Discontinuation at follow-up		−0.060 (−0.172 to 0.051)	.289		0.017 (−0.104 to 0.138)	.785		−0.015 (−0.148 to 0.117)	.820
New CS use		0.026 (−0.026 to 0.077)	.328		**0.066** (**0.009 to 0.122)**	.**022**		0.055 (−0.007 to 0.118)	.084
CS use at T1 and T2		**−0.224** (**−0.392 to −0.055)**	.**009**		−0.165 (−0.348 to 0.018)	.077		−0.169 (−0.360 to 0.022)	.082
Local CS use	77 018			66 681			57 228		
Discontinuation at follow-up		−0.003 (−0.034 to 0.027)	.836		−0.007 (−0.026 to 0.039)	.684		0.002 (−0.034 to 0.039)	.897
New CS use		**0.059** (**0.033 to 0.084)**	**<**.**001**		**0.048** (**0.021 to 0.075)**	.**001**		**0.037** (**0.006 to 0.067)**	.**017**
CS use at T1 and T2		0.010 (−0.023 to 0.042)	.548		**0.039** (**0.005 to 0.074)**	.**027**		0.023 (−0.018 to 0.065)	.265

Outcome of linear regression analyses on the outcome weight change in kg/year with the use of corticosteroids as the predicting variable. Model A gives the outcome of the crude analyses. Model B includes the covariates age, sex, baseline BMI, native Dutch ethnicity, educational level, smoking status, and alcohol use. Model C includes the covariates of model B, in addition to comorbidities (ie, cardiovascular diseases, asthma or chronic obstructive pulmonary disease, cancer, type 1 and type 2 diabetes mellitus). Bold indicates statistically significant (*P* < .05).

“Any CS use”: either systemic CS use or local CS use.

“Discontinuation at follow-up”: CS use reported at T1, but not at T2.

“New CS use”: CS use only reported at T2, not at T1.

Abbreviations: CS, corticosteroid; T1, baseline assessment; T2, second assessment.

Regarding WC changes (Table 4), we saw significant associations with new any CS use, and in new systemic CS use, and continued systemic CS use. After adjusting for covariates, we saw a significant association of discontinuation of CS use (ie, individuals reporting CS use at T1, but not T2) with WC change (β −.078 cm/year, *P* = .028), new any CS use (β .049 cm/year, *P* = .041), and new use of systemic CS (β .200 cm/year, *P* < .001).

**Table 4.** Linear regression estimates for waist circumference change between T1 and T2

	Model A—crude analyses			Model B—baseline characteristics			Model C—comorbidities		
	N	beta (95% CI)	*P* value	N	beta (95% CI)	*P* value	N	beta (95% CI)	*P* value
Any CS use	78 456			68 020			58 314		
Discontinuation at follow-up		**−0.064 (−0.122 to −0.005)**	.**034**		−0.055 (−0.118 to 0.009)	.094		**−0.078** (**−0.148 to −0.008)**	.**028**
New CS use		**0.045** (**0.006 to 0.084)**	.**023**		**0.044** (**0.001 to 0.087)**	.**044**		**0.049** (**0.002 to 0.096)**	.**041**
CS use at T1 and T2		0.043 (−0.008 to 0.093)	.097		0.045 (−0.010 to 0.100)	.107		0.025 (−0.040 to 0.090)	.455
Systemic CS use	63 530			55 035			47 427		
Discontinuation at follow-up		−0.076 (−0.254 to 0.101)	.400		−0.042 (−0.238 to 0.154)	.673		−0.105 (−0.318 to 0.108)	.334
New CS use		**0.165** (**0.083 to 0.246)**	**<**.**001**		**0.186** (**0.096 to 0.277)**	**<**.**001**		**0.200** (**0.099 to 0.302)**	**<**.**001**
CS use at T1 and T2		−0.219 (−0.485 to 0.046)	.105		**−0.302** (**−0.593 to −0.011)**	.**042**		−0.249 (−0.554 to 0.055)	.109
Local CS use	79 223			68 724			58 915		
Discontinuation at follow-up		−0.012 (−0.061 to 0.036)	.623		−0.005 (−0.057 to 0.048)	.867		−0.020 (−0.079 to 0.039)	.512
New CS use		0.038 (−0.002 to 0.079)	.063		0.035 (−0.009 to 0.079)	.123		0.036 (−0.013 to 0.085)	.152
CS use at T1 and T2		0.045 (−0.007 to 0.096)	.091		0.048 (−0.008 to 0.105)	.093		0.026 (−0.041 to 0.093)	.446

Outcome of linear regression analyses on the outcome waist circumference change in cm/year with the use of corticosteroids as the predicting variable. Model A gives the outcome of the crude analyses. Model B includes the covariates age, sex, baseline BMI, native Dutch ethnicity, educational level, smoking status, and alcohol use. Model C includes the covariates of model B, in addition to comorbidities (ie, cardiovascular diseases, asthma or chronic obstructive pulmonary disease, cancer, diabetes mellitus). Bold indicates statistically significant (*P* < .05).

“Any CS use”: either systemic CS use or local CS use.

“Discontinuation at follow-up”: CS use reported at T1, but not at T2.

“New CS use”: CS use only reported at T2, not at T1.

Abbreviations: CS, corticosteroid; T1, baseline assessment; T2, second assessment.

### Sensitivity Analyses

In the stratified analyses, we observed that the significant associations between CS use and changes in weight and WC occurred only in female participants, but not in male participants (Supplementary Tables S1 and S2 (16)). Compared to nonusers, we saw a significant increase in weight in female participants with new CS use at T2, continued use of CS, as well as new systemic CS use, new local CS use at T2, and continued use of local CS (β .053 kg/year, *P* = .004, β .051 kg/year, *P* = .049, β .089 kg/year, *P* = .022, β .058 kg/year, *P* = .003, and β .058 kg/year, *P* = .028 respectively). Furthermore, in female individuals there was a trend toward weight loss in individuals with continued systemic CS use compared to nonusers (β −.238 kg/year, *P* = .062). For WC, new systemic CS use was associated with higher WC increase in both male and female participants.

In the stratified analyses based on BMI class, we observed that there were only significant changes in weight and WC within the individuals with overweight or obesity, and not in those with a BMI <25 kg/m^2^ (Supplementary Tables S3 and S4 (16)). For weight change and WC change, we observed no significant associations in individuals with BMI <25 kg/m^2^. In individuals with overweight and obesity, however, discontinuation of CS use was associated with weight loss compared to nonusers (β −.090 kg/year, *P* = .006). New CS use and new local CS use was associated in trend with weight gain compared to nonusers (β .043 kg/year, *P* = .052, and β .042 kg/year, *P* = .064 respectively). Continued systemic CS use was associated in trend with weight loss (β −.224 kg/year, *P* = .090). For changes in WC, discontinuation of CS use and discontinuation of systemic CS use was associated with decrease in WC (Supplementary Table S4 (16)), whereas new CS use and new systemic CS use was associated with increase in WC compared to nonusers among individuals with BMI ≥25 kg/m^2^.

### Short-Term Changes in Weight in Relation to CS Use

Next, we assessed whether CS use was associated with acute effects on body weight (Supplementary Table S5 (16)). To this end, we analyzed the effects of weight change per week in relation to use of CS in a subset of participants. For the crude analyses, we had data of 60 956 individuals. Individuals reporting the use of any CS at baseline (week 1) reported a small but significant weight gain (β .005 kg/week, *P* = .038) compared to nonusers. New CS use (ie, no CS use at week 1, but reporting CS use at any time during the follow-up period) during the 12-week follow-up period was associated with a small but significant weight loss per week (β −.010 kg/week, *P* = .010), whereas discontinuation of CS use was associated with weight gain compared to nonusers. Analyzing systemic CS use and local CS use separately showed that only local CS use was associated with changes in weight, but not systemic CS use.

## Discussion

In this longitudinal population-based cohort study we assessed the effect of local and systemic corticosteroid (CS) use and its effects on changes in weight and waist circumference (WC) over a period of almost 4 years. Notably, we found a very high percentage of CS users (23.8%) in this population-based cohort during the study period. Here, we showed significantly higher weight gain in individuals reporting new use of any CS at follow-up and new use of local CS compared to nonusers. The use of new systemic CS was associated with increasing WC compared to nonusers, whereas discontinuation of CS use at follow-up was associated with decreasing WC compared to nonusers.

Furthermore, we showed these associations particularly in individuals with overweight or obesity (BMI ≥25 kg/m^2^) and in female participants. Interestingly, these associations between weight gain and CS use were opposite in the short term.

In our study we demonstrated that new use of locally administered CS was associated with weight gain in the long term. To our knowledge, this is the first paper to report long-term effects of local CS on body weight and WC in adults. Previously, it has been shown in children that inhalation corticosteroid (ICS) use is associated with BMI increase per year, and that the BMI decreased per year in children that switched from ICS to monoclonal antibodies for treatment of asthma (12).

The findings in our study strengthen the notion that some local corticosteroids should not be considered as only locally acting. It is known that with ICS only a fraction of the dose reaches the lung (ie, 10%-50%), while most of the administered dose is deposited in the oropharynx, swallowed, and absorbed through the gastrointestinal tract, after which it can exert its systemic effects (17). Furthermore, ICS are also absorbed across the lung and systemically deposited via the pulmonary circulation (18). Not only pharmacokinetically, but also pharmacodynamically there is a growing body of literature that local CS have systemic effects. In ICS users, for example, the hypothalamic-pituitary-adrenal (HPA) axis is suppressed, with lower overall plasma cortisol levels, and lower peak cortisol levels compared to nonusers, and these effects seem to be present even in individuals using the lowest dose of ICSs (11). Moreover, a meta-analysis by Broersen et al (10) shows that local CS (ie, inhalation, topical, and nasal) are associated with the development of adrenal insufficiency. This is supported by a previously published study from our group, which also shows subtle HPA-axis suppression in individuals using local corticosteroids in the same cohort as the current study (19).

In contrast to previously published studies (20, 21), we did not find a significant association between increased weight and continued use of systemic CS. On the contrary, we found a trend toward weight loss and continued systemic CS use and a significant association between WC increase and the start of new systemic CS. This discrepancy may be due to the anabolic effects on (visceral) adipose tissue of glucocorticoids, whereas the effects on lean mass may be more catabolic with subsequent muscle atrophy, altogether resulting in weight loss but with concomitant increases in waist circumference.

Yet, another possible explanation could be that we are investigating a group of individuals with such a severe illness that intensive prolonged therapy with CS was necessary. In clinical practice, continued high-dose CS use over years is rarely advised and all guidelines advise to taper this as soon as possible. Thus, the changes in these anthropometrics could be explained by the catabolic effects of underlying somatic disease for which prolonged systemic CS treatment is needed. Furthermore, prolonged treatment with high doses of glucocorticoids itself can lead to uncontrolled hyperglycemia, severe infections, and osteoporosis, all of which may contribute to weight. Finally, the number of cases (n = 167, Table 2) was relatively small. Therefore, these findings should be interpreted with caution.

Interestingly, we saw opposite effects of initiation and discontinuation of CS in the short-term analyses. We observed that initiation of local CS was associated with weight loss over the 12-week follow-up period, while discontinuation of local CS was associated with weight gain. A possible explanation for this finding could be that, in the short term, the catabolic effects of glucocorticoid action outweigh their anabolic effects, leading to a net reduction in body weight. It is likely that the weight gain associated with corticosteroid use emerges only after prolonged elevated glucocorticoid action (22). Additionally, the underlying disease for which corticosteroids are prescribed may contribute to weight loss, particularly shortly after corticosteroid initiation, due to catabolic impact of, for example, inflammatory conditions on the body. Conversely, the weight gain observed following corticosteroid discontinuation may reflect weight regain after initial weight loss caused by the disease.

In our study, we found differential results when stratifying the analyses by sex. For male participants, we found a nonsignificant association between weight change and the use of CS. In female participants, however, association between CS use with changes in weight was more pronounced.

Interestingly, we previously also found that local CS use and the association with metabolic syndrome at a cross-sectional level was also more pronounced in women (8). Side effects of CS use are also more frequently seen in women compared to men (23, 24). And a previous study assessing long-term systemic CS use found that women were at higher risk of pronounced weight gain (20). These differing findings between male and female individuals may be explained by previously observed sex differences in adherence to medication or sex differences in glucocorticoid sensitivity or metabolism. We know from previous studies that women tend to be more treatment adherent to the use of ICS (25). Also, in vivo glucocorticoid (GC) sensitivity assays show that women have higher GC sensitivity compared to men (26), and that women using oral contraceptives have higher GC sensitivity compared to women who do not use oral contraceptives (27). Furthermore, it is suggested that GC negative feedback seems to be lower in women compared to men (28), and women are exposed to different social circumstances, such as past trauma, that may also affect the setpoint of the HPA-axis (29). These findings suggest a role of sex hormones in GC sensitivity in humans. Additionally, it is important to consider that factors such as education levels, smoking, and alcohol use may significantly influence effect size, as demonstrated by the attenuation of the association between CS use and changes in weight and WC when these variables are accounted for.

Lastly, we found differing results stratifying the analyses by BMI class (ie, <25 kg/m^2^ and ≥25 kg/m^2^). In individuals with normal weight or underweight, we found no significant associations between CS use and changes in weight or WC. In individuals with overweight/obesity we did find an association with both weight loss and decrease in WC, and discontinuation of CS use. New (local) CS use was associated in trend with higher weight gain in this subgroup. New systemic CS use at T2 was associated with increase in WC in individuals with overweight/obesity. The exact mechanism between GC use and its adverse effects in relation to BMI is not fully understood, although there is some indication that GC action is related to overweight and obesity. Previous studies have shown that glucocorticoid receptor (GR) sensitivity is related to body weight and metabolic profile (30, 31). Individuals with GR polymorphisms associated with a relatively increased GC sensitivity may predispose to obesity. Cross-sectionally, CS use was indeed found to be associated with both higher BMI and higher presence of metabolic syndrome in individuals with these GR hypersensitivity polymorphisms (32). Also, hair GC level, which is thought to be a marker of long-term endogenous GC exposure, was positively associated with BMI and waist circumference in non-CS users (33-35).

One of the strengths of our study is that it concerns a large population-based cohort and it prospectively assesses the effects of CS use on measures of obesity (ie, weight and waist circumference) in a large population-based sample with a relatively long follow-up period. An important limitation of the study is the possibility of recall bias, as CS medication was self-reported using questionnaires at T2. Unfortunately, we did not have data regarding intermittent CS use between T1 and T2 follow-up. Another limitation relates to the reliance on self-reported weight and CS data within our study, which could potentially introduce measurement error into our study. However, studies have shown that the use of self-reported weight measures and medication use are valid and can be used in large epidemiological studies (36-38), and baseline medication use was thoroughly assessed and checked by a study nurse during the T1 visit. Another limitation in the analyses of the long-term weight gain was the unavailability of data on meal frequency and physical activity, limiting the comparability of the weight gain analyses with the COVID-19 subcohort where these variables were assessed. Similarly, while thyroid dysfunctions are prevalent and could impact weight outcomes, we only had baseline thyroid function data for a subset of participants, and these were not included in the analyses. We also assumed that individuals with known thyroid dysfunctions were adequately treated, minimizing potential effects on weight trajectories. Menopausal status is another important factor that may influence weight gain due to hormonal changes in perimenopause and postmenopausal periods. However, we only had data on menopausal status at baseline, limiting our ability to assess its potential impact over the follow-up period. Additionally, no data on the history of psychological trauma was available, which might confound our results as past trauma and presence of posttraumatic stress disorder symptoms have been associated with an increased risk of weight gain (39, 40). Finally, the associations that we have found regarding CS use and changes in weight and WC do not prove causation, and therefore more studies are needed to elucidate causality.

## Conclusion

In summary, we find that corticosteroid use is highly prevalent (23.8%) in this population-based cohort study, and we show that both systemic and in particular also locally administered corticosteroid use is associated with long-term changes in weight and waist circumference. Overall, corticosteroid users have significantly more weight gain and increase in waist circumference compared to nonusers. Our results highlight the importance of critically evaluating chronic use of both systemic and local corticosteroids, as discontinuation may have a beneficial effect on measures of obesity in selected patients.

## Data Availability

Restrictions apply to the availability of some or all data generated or analyzed during this study to preserve patient confidentiality or because they were used under license. The corresponding author will on request detail the restrictions and any conditions under which access to some data may be provided.

## Abbreviations

BMI: body mass index
CS: corticosteroid
COPD: chronic obstructive pulmonary disease
CVD: cardiovascular disease
GC: glucocorticoid
HPA: hypothalamic-pituitary-adrenal
WC: waist circumference
